# Daily Dietary Sodium Intake Among Clinical Trial Participants Recruited From a University Health System or a Federally Qualified Health Center: Secondary Analysis of Baseline Participant Characteristics

**DOI:** 10.2196/71343

**Published:** 2025-09-25

**Authors:** Gabriella V Rubick, Michael P Dorsch, Scott L Hummel, Tanima Basu, Evan Luff, Kimberly Warden, Michael Giacalone, Sarah Bailey, Mark W Newman, Lesli E Skolarus, Brahmajee K Nallamothu, Jessica R Golbus

**Affiliations:** 1 Department of Internal Medicine, University of Michigan–Ann Arbor, 1500 E Medical Center Dr., Ann Arbor, MI, 48109, United States, 1 7349364000; 2 Department of Clinical Pharmacy, College of Pharmacy, University of Michigan–Ann Arbor, Ann Arbor, MI, United States; 3 Division of Cardiovascular Medicine, Department of Internal Medicine, University of Michigan–Ann Arbor, Ann Arbor, MI, United States; 4 Department of Internal Medicine, Section of Cardiology, VA Ann Arbor Healthcare System, Ann Arbor, MI, United States; 5 Hamilton Community Health Network, Flint, MI, United States; 6 Bridges into the Future, Flint, MI, United States; 7 School of Information and Department of Electrical Engineering and Computer Science, University of Michigan–Ann Arbor, Ann Arbor, MI, United States; 8 Department of Neurology, Stroke and Vascular Neurology, Northwestern Medicine, Chicago, IL, United States; 9 Michigan Integrated Center for Health Analytics and Medical Prediction, University of Michigan–Ann Arbor, Ann Arbor, MI, United States

**Keywords:** sodium consumption, clinical trial, hypertension, cardiovascular diseases, blood pressure, dietary sodium, sociodemographic factors

## Abstract

**Background:**

Efforts to improve diversity in clinical trials often prioritize recruitment based on broad demographic factors. This approach may overlook the influence of community context and health-related social needs on health behaviors, including sodium intake, a key modifiable risk factor for hypertension and cardiovascular disease.

**Objective:**

This study aims to assess the impact of enrollment site, sociodemographic factors, and health-related social needs on baseline dietary sodium intake among participants in a mobile health clinical trial aimed at lowering blood pressure.

**Methods:**

The myBPmyLife study is a prospective, randomized controlled trial evaluating a mobile health intervention to lower blood pressure through increased physical activity and lower sodium food choices. Participants with hypertension were recruited from a university health system and a federally qualified health center (FQHC). All participants completed a validated sodium screener at enrollment. Sociodemographic data and health-related social needs were self-reported. Univariable and multivariable linear regression models were used to evaluate the associations between sodium intake and participant characteristics. This analysis presents a cross-sectional examination of the baseline characteristics of participants enrolled in the myBPmyLife study.

**Results:**

Among 600 included participants, 96 (16.0%) were from the FQHC. Mean age was 60.1 (SD 13.5) years; 48.2% (289/600) were women, and 13.0% (78/600) were Black. FQHC participants were significantly younger (mean age 47.9, SD 11.1 vs 62.5, SD 12.7 years), more likely to be Black (43/96, 44.8% vs 35/504, 6.9%), and 8.5 times more likely to have difficulty paying for their health-related social needs. Mean baseline sodium intake was 3082.3 (SD 1072.5) mg/day, with 85.5% (513/600) of participants exceeding the World Health Organization’s recommended daily sodium limit. Baseline sodium intake was significantly higher for FQHC participants (mean difference 381.1, SD 1064.2 mg/d; 95% CI 84.5-677.7; *P*=.01), men (mean difference 543.9, SD 1038.3 mg/d; 95% CI 377.3-710.5; *P*<.001), Black participants (mean difference 442.5, SD 1043.4 mg/d; 95% CI 119.7-765.3; *P*=.008) and those with difficulty affording basic needs (mean difference 338.1, SD 1066.7 mg/d; 95% CI 95.2-581.0; *P*=.02). Sodium intake was lower in older participants (−196.4 mg/d per 10 years; 95% CI −258.0 to −134.9; *P*<.001). In a multivariable analysis, age, gender, and race remained independently associated with sodium intake, while differences by site and health-related social needs were not statistically significant.

**Conclusions:**

Differences in sodium intake were observed across sociodemographic groups. While the enrollment site was not independently associated with sodium intake after adjustment, it played a role in shaping the participant population, evidenced by the differences in demographics and health-related social needs among participants based on enrollment site. These findings underscore the importance of recruiting from distinct clinical settings to capture a range of contextual factors that influence health behaviors. Clinical trials aiming for representativeness should consider both individual- and community-level factors during recruitment to more accurately inform interventions and health outcomes.

## Introduction

There is an increasing focus on recruiting diverse participants for clinical trials to ensure representative study populations [1-3]. These recruitment strategies mainly aim to include diverse populations based on standard demographic characteristics such as gender, race, age, and ethnicity. For instance, these factors have been linked to dietary sodium intake [4-11], a major contributor to hypertension and cardiovascular disease development [12,13]. However, concentrating solely on these broad participant characteristics of underrepresented groups may overlook the influence of community-level differences on health outcomes, as women, Black individuals, or older patients from one community may differ from those of similar backgrounds in another.

Although there is an increased understanding of how individual demographic factors influence health behaviors, a knowledge gap remains regarding how the context of recruitment settings and related community-level factors affect both participant traits and baseline health behaviors such as sodium intake in clinical trials. Thus, exploring differences between site characteristics and health outcomes can improve our understanding of population diversity and aid in developing targeted interventions.

This analysis contributes to this topic by analyzing a cohort of patients with hypertension recruited into the myBPmyLife clinical trial. This trial is well suited to investigate this question, as it included community members from 2 distinct clinical settings: a large, academic university health system and a federally qualified health center (FQHC) clinic. Notable population-level differences were observed between the 2 clinical settings during the trial, including the requirement for study team resources during recruitment and health-related social needs [14]. By analyzing sodium intake at baseline in a diverse participant group, we aimed to understand the impact of demographic characteristics, health-related social needs, and enrollment site on dietary sodium consumption within a mobile health (mHealth) clinical trial.

## Methods

### Study Design

The myBPmyLife study is a prospective, randomized controlled, remotely administered trial (ClinicalTrials.gov NCT05154929). Participants with hypertension were recruited from Michigan Medicine in Ann Arbor, MI, and the Hamilton Community Health Network in Flint, MI. Participants were randomly assigned to the intervention group, which received an mHealth intervention promoting increased physical activity and lower sodium food choices, or to the control group. This analysis reports on a secondary study objective: understanding sodium intake in hypertensive individuals with diverse sociodemographic characteristics. It is a baseline analysis of data collected from participants prior to randomization, and as such, all participants, regardless of study group, are included. The full study protocol and results for the primary trial outcomes have been published [15,16]. The authors are solely responsible for the design and conduct of the study, all study analyses, and drafting and editing of the paper.

### Eligibility

The study was designed to recruit patients with self-reported hypertension who could safely be physically active and reduce their sodium intake. Patients were considered eligible if they were 18 years of age or older with self-reported hypertension, had no hypertensive medication changes in the prior 4 weeks, and had a compatible smartphone. Exclusion criteria included contraindications to physical activity or sodium restriction, a secondary cause of hypertension, and a sodium consumption of <1500 mg/day as estimated by the NutritionQuest Sodium Screener (NutritionQuest), which was completed by all potential participants following informed consent. Full inclusion and exclusion criteria are available in Table S1 in Multimedia Appendix 1. In addition, 2 participants listed their gender as “other.” Due to the extremely limited sample size within this category and the resultant inability to perform meaningful statistical analyses or draw robust conclusions regarding this subgroup, these participants were excluded from this cross-sectional analysis.

### Trial Procedures

The myBPmyLife study launched in December 2021. Participants were recruited from Michigan Medicine and the Hamilton Community Health Network. Michigan Medicine is a large quaternary referral center. Its facilities are primarily located within Ann Arbor, with a median household income of US $84,245 and a 13.8% poverty rate. In contrast, the Hamilton Community Health Network is an FQHC network in Flint, MI, and delivers primary care services. Flint, MI, has a median household income of US $35,451, with a poverty rate of 33.3%, over double the poverty rate of Ann Arbor [17].

Recruitment varied by study site. The *International Classification of Diseases, Tenth Revision* (*ICD‐10*) code I10 identified participants at both sites. Potentially eligible participants were recruited using weekly emails and text messages. Participants were preferentially recruited if they had an upcoming appointment at the Hamilton Community Health Network or with a Michigan Medicine primary care physician. The study intervention focused on delivering push notifications tailored to the participants to promote physical activity and the selection of lower-sodium food choices. The study mobile app provided feedback on participants’ progress toward these goals.

### Data Collection and Study End Points

All participants were required to download the MyDataHelps mobile app, a commercially available research app developed by CareEvolution. After completing the informed consent process, participants used the app to complete the NutritionQuest Sodium Screener. Participants with an estimated sodium intake of less than 1500 mg/day were excluded from the study. The NutritionQuest Sodium Screener is a 26-item screener developed from 24-hour recall data from adults in the National Health and Nutrition Examination Survey (NHANES) from 2007 to 2008. For this screener, foods contributing to 80% of sodium intake are included, with survey respondents asked to rate the frequency at which they consume each of the foods over the prior 24 hours. This sodium screener has previously been validated in 2 studies as compared to 24-hour dietary recall with good correlation noted [18,19].

Patient-reported surveys were used to obtain sociodemographic characteristics, health-related social needs, and medical comorbidities. Specifically, health-related social needs were determined by the question, “How hard is it for you to pay for the very basics like food, housing, medical care, and heating?” Audio and video calls were used to collect medication data and to confirm sociodemographic information when necessary due to technical issues.

### Statistical Analysis

Baseline sociodemographic characteristics are described as means and SDs for continuous symmetric variables and medians with IQRs for skewed continuous variables. Categorical variables are presented as counts and percentages. We performed 2-tailed Student *t* tests to compare continuous variables and the chi-square test for categorical variables. Age and sodium intake were coded as continuous variables, with all other sociodemographic categories coded as categorical variables.

We performed a series of univariable and multivariable generalized linear models to estimate associations between key sociodemographic and clinical characteristics and sodium intake. Features within multivariable models were selected a priori based on clinical expertise and the available literature and included clinical setting, age, gender, race, and health-related social needs. Subsequently, stratified models were developed for each clinical setting. The confidence level for the lower and upper confidence limits was set at 95%. Statistical analysis was completed using Statistical Analysis System version 14.2 (SAS Institute).

### Ethical Considerations

The University of Michigan Institutional Review Board (HUM00205845) approved the study. Informed consent was obtained for all participants after the nature and possible consequences of the study were explained, in compliance with local, institutional, and national regulations on research involving human participants. Informed consent was obtained by telephone, with the option to use videoconferencing as needed, with the consent form signed within the MyDataHelps mobile app. Study data were aggregated and published in a deidentified fashion. Incentives for participation included a financial incentive of US $100 split over 2-month time points and the ability to keep the study-provided smartwatch and blood pressure cuff, contingent on survey completion and engagement with the mobile intervention for at least 2 months.

## Results

### Study Population

**Figure 1.**
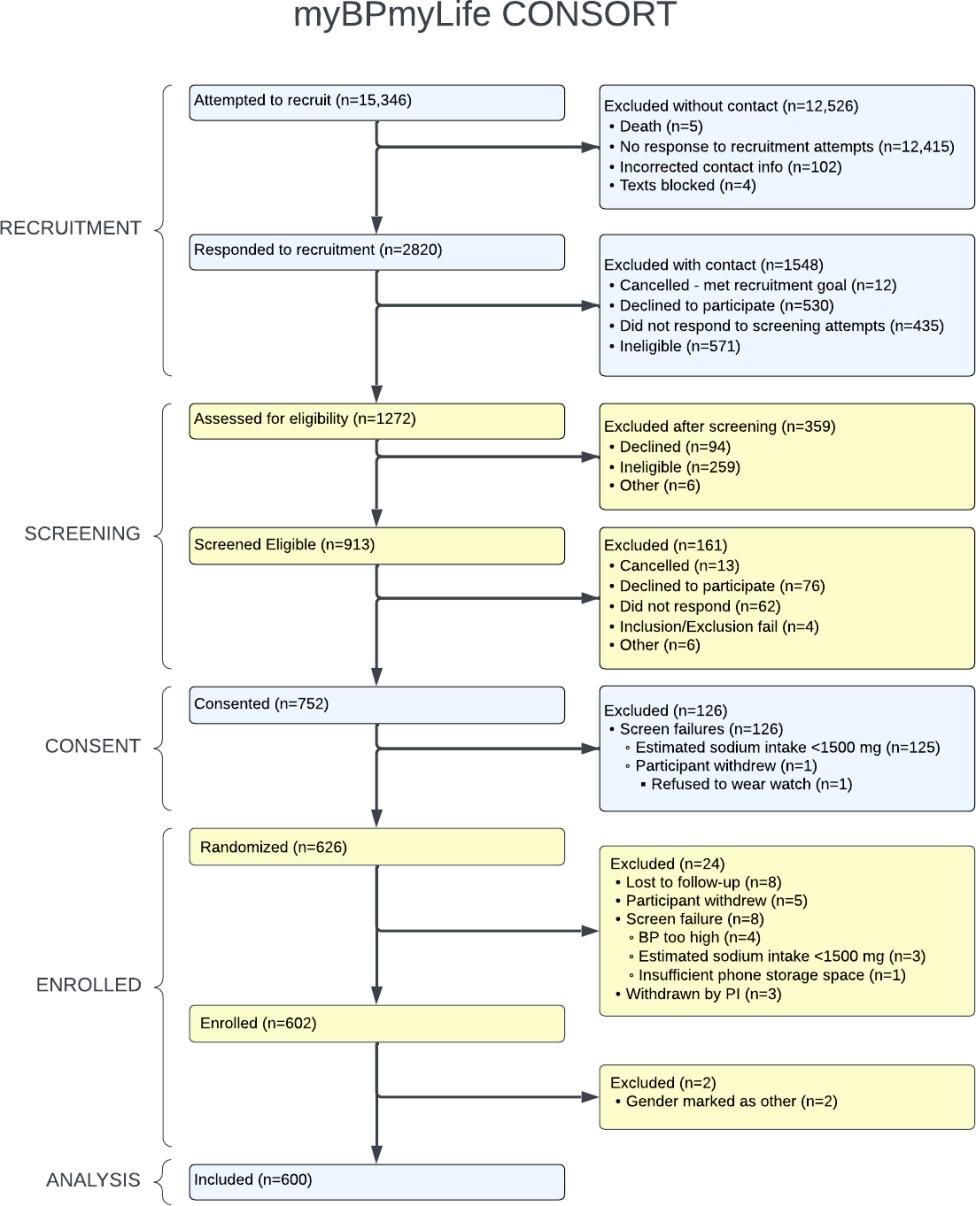
Consolidated Standards of Reporting Trials (CONSORT) diagram for the myBPmyLife trial.

Between December 2021 and July 2023, 752 patients consented to participate in the myBPmyLife study. A CONSORT (Consolidated Standards of Reporting Trials) diagram for this study can be found in Figure 1. Of these 752 patients, 150 were ineligible, with 128 excluded for having a baseline sodium intake of <1500 mg/day, 13 from the FQHC, and 115 from the university health system. Participants who were ineligible due to a sodium intake of <1500 mg/day were more likely to be women (89/128; 69.5%), White (98/128; 76.6%), and not Hispanic (125/128; 97.7%) (Table S2 in Multimedia Appendix 1). After exclusions, 602 participants enrolled in the study. Two participants reported their gender as “other” and were excluded from subsequent analyses due to the limited sample size. Of the remaining 600 participants, 96/600 (16.0%) were from the FQHC. Participants’ mean age was 60.1 years (SD 13.5), 289/600 (48.2%) self-identified as women, and 78/600 (13.0%) self-identified as Black. With regard to comorbidities, 28/600 (4.7%) participants reported a history of chronic kidney disease, 267/600 (44.5%) reported a history of hyperlipidemia, 118/600 (19.7%) reported a history of depression, 43/600 (71.7%) reported a history of atrial fibrillation or atrial flutter, 16/600 (2.5%) reported a history of stroke, and 134/600 (22.3%) reported a history of diabetes. FQHC participants were significantly younger than university health system participants (47.9, SD 11.1 vs 62.5, SD 12.7 years) and more likely to be Black (43/96, 44.8% vs 35/504, 6.9%) and women (61/96, 63.5% vs 228/504, 45.2%) (Table 1). In addition, 56.3% (54/96) of FQHC participants reported at least some difficulty paying for “the very basics like food, housing, medical care, and heating” compared to 6.6% (33/504) of the university health system participants.

**Table 1.** Participant characteristics stratified by study site.

	University health system (n=504)	FQHC^a^ (n=96)	Total (N=600)	*P* value
Age (years), mean (SD)	62.5 (12.7)	47.9 (11.1)	60.1 (13.5)	<.001
Gender, n (%)				.001
Women	228 (45.2)	61 (63.5)	289 (48.2)	
Men	276 (54.8)	35 (36.5)	311 (51.8)	
Race, n (%)				<.001
White	402 (79.8)	44 (45.8)	446 (74.3)	
Black	35 (6.9)	43 (44.8)	78 (13.0)	
Other^b^	67 (13.3)	9 (9.4)	76 (12.7)	
Ethnicity, n (%)				.94
Hispanic	15 (3.0)	3 (3.1)	18 (3.0)	
Health-related social needs question, n (%)				
How hard is it for you to pay for the very basics such as food, housing, medical care, and heating?				<.001
Not hard at all	471 (93.5)	42 (43.8)	513 (85.5)	
Somewhat hard	31 (6.2)	47 (49)	78 (13)	
Very hard	2 (0.4)	7 (7.3)	9 (1.5)	

a FQHC: federally qualified health center.

b Other: Asian, American Indian, Native Hawaiian, Other Pacific Islander, multiple, other, or refused to answer.

### Factors Associated With Elevated Sodium Intake

Most study participants consumed more than the World Health Organization–recommended 2000 mg of sodium daily (513/600, 85.5%) [20]. The study cohort’s mean sodium intake was 3082.3 (SD 1072.5) mg/day, ranging from 1503 to 8886 mg/day (Table 2; Figure S1 in Multimedia Appendix 1). Sodium intake was 3021.4 (SD 987.5) mg/day for university health system participants compared to 3402.5 (SD 1402.2) mg/day for FQHC participants.

Sodium intake varied across sociodemographic characteristics. Notably, sodium intake was lower in older participants compared to younger participants. Sodium intake was significantly higher in men compared to women and in Black compared to White participants. Finally, participants who reported it was “somewhat hard” or “very hard” to pay for health-related social needs such as food, housing, medical care, and heating consumed significantly more dietary sodium than those who found it “not hard” to pay for those items(Table 2).

**Table 2.** Univariable analysis of sodium intake based on sociodemographic characteristics and site of enrollment.

Characteristics		Mean	95% CI	*P* value
Clinical setting				
	University Health System	3021.4 (987.5)	2934.9 to 3107.8	0.01
	FQHC^a^	3402.5 (1402.2)	3118.4 to 3686.6	N/A^b^
Age (per 10 years)		−196.4 (768.9)	-258 to -134.9	<.001
Gender				
	Women	2800.4 (985.3)	2686.4 to 2914.5	<.001
	Men	3344.3 (1085.2)	3223.2 to 3465.4	N/A
Race				
	White	3021.8 (974.3)	2931.1 to 3112.5	N/A
	Black	3464.3 (1376.3)	3154 to 3774.6	0.008
	Other^c^	3045.6 (1197.9)	2771.9 to 3319.4	0.92
Ethnicity				
	Non-Hispanic	3082.9 (1067.6)	3002.2 to 3176.7	N/A
	Hispanic	3064.9 (1255.6)	2440.5 to 3689.3	0.92
Health-Related Social Needs Question				
	How hard is it for you to pay for the very basics like food, housing, medical care, and heating?			
	Somewhat hard/very hard	3371.4 (1313.9)	3091.4 to 3651.5	0.02
	Not hard	3033.3 (1019.3)	2944.9 to 3121.7	N/A

a FQHC: federally qualified health center.

b Not applicable.

c Asian, American Indian, Native Hawaiian, Other Pacific Islander, multiple, other, or refused to answer.

A multivariable model was also constructed to understand the relative contributions of sociodemographic characteristics to dietary sodium intake (Table 3). As in the univariable analyses, sodium intake was lower in older individuals and was higher in men and Black participants. However, there was no significant difference in sodium intake between clinical settings or health-related social needs after accounting for these other factors (Table 3).

**Table 3.** Multivariate analysis of sodium intake for the entire cohort and distributed by clinical setting.

	Entire cohort				University health system				FQHC^a^			
	Estimate	LCL^b^	UCL^c^	*P* value	Estimate	LCL	UCL	*P* value	Estimate	LCL	UCL	*P* value
Age (per 10 years)	−153.5	−221	−86.9	<.001	−104.4	−172.4	−36.5	.003	−450.8	−686.8	−214.8	<.001
How hard is it for you to pay for the very basics such as food, housing, medical care, and heating?												
Not hard	−172.2	−441.9	97.4	.21	−296.6	−641.2	48.1	.092	−181.9	−705.3	341.4	.50
Somewhat hard or very hard	—^d^	—	—	—	—	—	—	—	—	—	—	—
Race												
Black	311.8	44.4	579.2	.02	268.9	−61.8	599.6	.11	413.3	−121.2	947.9	.13
Other^e^	−134.1	−382.2	113.9	.29	−77.0	−325.3	171.2	.54	−244.8	−1213.1	723.6	.62
White	—	—	—	—	—	—	—	—	—	—	—	—
Ethnicity												
Non-Hispanic	164.5	−313.3	642.3	.50	47.0	−442.2	536.1	.85	516.7	−1037.4	2070.7	.52
Hispanic	—	—	—	—	—	—	—	—	—	—	—	—
Gender												
Women	−565.9	−731.0	−400.9	<.001	−529.3	−698.1	−360.6	<.001	−783.8	−1315.5	−252.0	.005
Men	—	—	—	—	—	—	—	—	—	—	—	—
Clinical setting												
FQHC	51.9	−231.5	335.2	.72	—	—	—	—	—	—	—	—
University health system	—	—	—	—	—	—	—	—	—	—	—	—

a FQHC: federally qualified health center.

b LCL: lower confidence limit.

c UCL: upper confidence limit.

d Not applicable.

e Asian, American Indian, Native Hawaiian, Other Pacific Islander, multiple, other, or refused to answer.

## Discussion

### Principal Findings

Understanding the impact of demographic factors, health-related social needs, and enrollment sites on outcomes in mHealth clinical trials is relevant to ensuring enrollment of diverse patient populations. Ultimately, ensuring the recruitment of representative cohorts is crucial to shaping accurate health data for future clinical care. In this contemporary trial, sodium intake was significantly higher in younger participants, men, Black participants, and those who had difficulty paying for health-related social needs. Many of these differences in sodium intake are both statistically and clinically significant, as data show that reducing daily sodium intake by just 400 mg significantly impacts population-level cardiovascular events and mortality [21]. In addition, we found significant differences in sodium intake between our 2 study sites. However, the impact of the enrollment site on sodium intake was minimized after accounting for demographic characteristics and health-related social needs, indicating that these factors play a crucial role in study outcomes. Ultimately, this study highlights the unique influence of recruitment settings on clinical trial participant diversity and baseline health behaviors, specifically sodium intake. While the enrollment site was not independently associated with sodium intake after adjusting for individual factors, it serves as a crucial contextual indicator for underlying demographic and socioeconomic influences on health behaviors. This underscores the importance of considering community-specific factors and health-related social needs beyond broad demographic categories when recruiting for trials.

### Comparison to Prior Work

In the multivariable models of this study, only age and sex remained significantly associated with sodium intake. These findings are consistent with prior studies, including data from NHANES for the years 1999‐2016, which have shown that younger adults and men tend to consume more sodium, in part due to differing dietary preferences and behavioral patterns [4-8,22]. While race and health-related social needs were both associated with sodium intake in unadjusted analyses, these associations attenuated in adjusted models, suggesting that their effects may be mediated or confounded by other covariates. It is also possible that our binary measure of health-related social needs did not fully capture the complexity of socioeconomic disadvantage. Notably, 1999‐2016 NHANES data also do not show a difference in sodium intake by income level, but do note higher sodium intake in White individuals compared to Black individuals, which contrasts with our findings. Despite the lack of independent statistical significance in our adjusted models, it is possible that structural and contextual differences, such as those captured by study site, still play a meaningful role in shaping dietary behavior and access to healthy food, particularly given the large disparities observed in unadjusted comparisons.

Research into how community context affects health outcomes, known as “neighborhood effects on health,” is a growing area of investigation [23]. It examines how factors such as poverty, walkability, and food accessibility affect health outcomes in a specific community and, from that, what location-specific interventions are optimal for promoting population health. Many studies show that neighborhood or community context adjustments often diminish racial and ethnic differences in health research [24-27]. Notably, in our study, 56.3% (54/96) of the FQHC cohort reported at least some difficulty paying for necessities compared to 6.6% (33/504) of university health system participants. In Flint, MI, where the FQHC is located, a large part of the community is a food desert, defined as a low-income area with at least 500 people, or 33% of the population, living more than 1 mile from the nearest supermarket [28]. In light of this, it is not surprising that individuals from the FQHC, whose primary mission is to provide care for all individuals regardless of their ability to pay for services [29], were more likely to experience difficulty in paying for health-related social needs compared to individuals from the university health system located in a comparatively more affluent area. These community factors and decreased access to healthy food options also likely affected sodium intake in FQHC participants.

### Study Strengths and Limitations

This study has multiple strengths. First, the enrollment of participants from a large university health system and an FQHC significantly augmented the diversity of our patient population. These varied enrollment sites allowed for a nuanced analysis of the role of clinical and community-based factors in health outcomes. Second, the study design, which did not necessitate in-person visits, expanded the participant pool beyond those traditionally enrolled in clinical trials. Finally, there were high response rates as baseline surveys were completed with the study coordinators.

There are several relevant limitations to this study as well. First, participants were required to own a smartphone capable of downloading study software, excluding 22 potential participants. While smartphone ownership in the United States is high (approximately 90%) [30], this criterion may still bias the study population toward more technologically resourced individuals. Future trials may consider expanding study participation through loaner phones. Second, participants were required to consume >1500 mg/day of sodium to be eligible for the study, excluding individuals who are already meeting the suggested dietary sodium goals. Third, key data, including sodium intake and sociodemographic characteristics, were self-reported. This introduces the possibility of recall bias and systematic bias, both intake-related and person-specific, as previously noted in food frequency questionnaires [31]. To mitigate this, we used a validated sodium screener. However, this screener also requires subjective reporting of dietary intake, and previous studies have noted that women tend to underreport their intake. This may account for the gender-specific differences observed in sodium intake [32]. Objective measures such as 24-hour urinary sodium or medical record review could further enhance data accuracy in future studies. Fourth, we did not adjust for total energy intake or BMI, which may partially explain higher sodium intake in certain subgroups due to greater caloric needs. However, the persistence of significant differences by race and health-related social needs suggests that other social and contextual factors may contribute to sodium intake. Fifth, we did not collect data on factors such as income, employment, insurance, or marital status, which could provide further insight into the relationship between socioeconomic factors and sodium intake. Finally, our study used a cross-sectional design, which precludes causal inference and the establishment of temporal relationships. Our findings demonstrate associations, but not causality; longitudinal studies are needed to explore causal pathways.

### Future Directions

Clinical trials aiming for representative populations must consider how site-level recruitment strategies shape participant characteristics and influence health behaviors. This study emphasizes the community-specific factors that significantly impact health-related outcomes and calls for mHealth researchers to also consider the community and clinical settings from which participants are recruited when considering diversity. In addition, future studies should collect comprehensive data on food environment and accessibility to further elucidate their impact on dietary behaviors and enhance our understanding of how these contextual factors influence trial participant characteristics and health outcomes. By doing so, we can move away from simplistic race, gender, and age-based comparisons for intervention and recruitment strategies and develop a more nuanced methodology that reflects the intricate interplay between community factors, health-related social needs, and health outcomes. This approach aligns with recent calls from the National Academies of Sciences to critically re-evaluate the use of race and ethnicity in biomedical research, advocating for a focus on underlying social and environmental determinants rather than using these categories as proxies for biological differences [33].

### Conclusions

In this clinical trial of an mHealth intervention, we observed significant differences in sodium intake among participants with variability across clinical sites and according to demographic characteristics and health-related social needs. The differences between the 2 clinical settings from which participants for this trial were recruited are limited examples, though they highlight striking differences between the groups. Although adjustment for demographic and health-related social needs minimized differences in sodium intake between sites, these broad stratifications do not fully account for the differences between these 2 communities. These findings highlight that the composition of trial populations, and thus trial outcomes, can be shaped by the recruitment setting.

## Supplementary material

10.2196/71343 Multimedia Appendix 1 Supplementary tables and figure, including full inclusion and exclusion criteria for the myBPmyLife trial, demographic characteristics of participants who were excluded due to a daily sodium intake of <1500 mg, and overall estimated dietary sodium intake (mg/day) distribution.

## Abbreviations

CONSORT: Consolidated Standards of Reporting Trials
FQHC: federally qualified health center
*ICD-10*: *International Classification of Diseases, Tenth Revision*
mHealth: mobile health
NHANES: National Health and Nutrition Examination Survey
