# Supplementary material for: Daily Dietary Sodium Intake Among Clinical Trial Participants Recruited From a University Health System or a Federally Qualified Health Center: Secondary Analysis of Baseline Participant Characteristics
Source: JMIR Cardio. 2025 Sep 25;9:e71343. doi: 10.2196/71343 (PMC12463386; doi:10.2196/71343)

**Table S1.** Full inclusion and exclusion criteria for the myBPmyLife trial.

| Inclusion Criteria |
| --- |
| - Self-reported history of hypertension and no hypertensive medication changes in the last 4 weeks |
| - A smartphone with a compatible Apple or Android operating system installed and able to download and use the myBPmyLife app including accepting all permissions. |
| - A valid email address |
| - Fluent in spoken and written English |
| - Signed written informed consent. (Note that each participant must be able to consent for themselves.) |
|  |
| Exclusion Criteria |
| - Contraindication to performing physical activity or following a sodium restriction diet. The participant must be able to walk and eat on their own without assistive devices. |
| - Unstable symptoms or markedly elevated BP at enrollment (defined as systolic BP >180 mmHg, diastolic BP >120 millimeters of mercury (mmHg)) |
| - Known secondary causes of hypertension (e.g., adrenal insufficiency, pheochromocytoma), heart failure, or end-stage renal disease |
| - Difficulty using an upper arm blood pressure cuff due to biceps size or end-stage renal disease or difficulty comfortably wearing a smartwatch |
| - Wrist too large to wear a smartwatch comfortably. |
| - Daily sodium intake less than 1500 mg/day as estimated by the sodium screener |
| - Currently pregnant or planning to become pregnant in the next six months |

**Table S2.** Demographic characteristics of participants who were excluded due to a daily sodium intake of <1500 mg. *Other: Asian, American Indian, Native Hawaiian, Other Pacific Islander, Multiple, Other, or Refused to answer.

|  | University Health System (N=115) | FQHC (N=13) | Total (N=128) |
| --- | --- | --- | --- |
| Age in years |  |  |  |
| Mean (SD) | 67.0 (10.7) | 53.3 (10.2) | 66.1 (11.4) |
|  |  |  |  |
| Gender |  |  |  |
| Women | 78 (67.8%) | 11 (84.6%) | 89 (69.5%) |
| Men | 36 (31.3%) | 2 (15.4%) | 38 (29.7%) |
| Other | 1 (0.9%) | 0 (0.0%) | 1 (0.8%) |
|  |  |  |  |
| Race |  |  |  |
| White | 89 (77.4%) | 9 (69.2%) | 98 (76.6%) |
| Black | 9 (7.8%) | 3 (23.1%) | 12 (9.4%) |
| Other/ Multiple* | 17 (14.8%) | 1 (7.7%) | 18 (14.0%) |
|  |  |  |  |
| Ethnicity |  |  |  |
| Not Hispanic | 113 (98.3%) | 12 (92.3%) | 125 (97.7%) |
| Hispanic | 2 (1.7%) | 1 (7.7%) | 3 (2.3%) |

**Figure S1.** Overall estimated dietary sodium intake (mg/day) distribution.


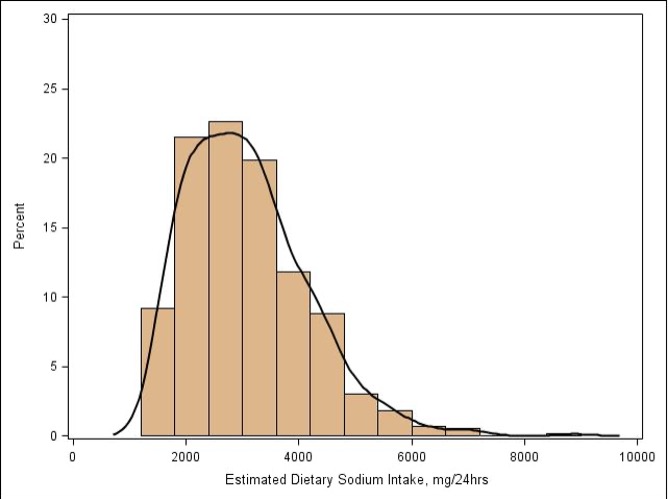

Supplement: Multimedia Appendix 1 [file cardio-v9-e71343-s001.docx]
